# Mediterranean Diet: The Need for Cross-Disciplinary Perspectives

**DOI:** 10.3390/ijerph18115687

**Published:** 2021-05-26

**Authors:** F. Xavier Medina

**Affiliations:** FoodLab & UNESCO Chair on Food, Culture and Development, Faculty of Health Sciences, Open University of Catalonia, 08018 Barcelona, Spain; fxmedina@uoc.edu

## 1. Introduction

The notion of the Mediterranean diet has progressively evolved over the past half a century, from a healthy (coronary) dietary pattern to a model of sustainable diet [1,2], passing through culture (cultural heritage) on the way [3,4]. This evolution has transformed the whole concept of the Mediterranean diet from strictly medical and nutritional positions to visions more closely linked with society, culture, lifestyles, economy, sustainability, and environment.

It is true that the centrality of the perspective related to health continues to be the main axis around which all other aspects rotate. However, we also have to admit that these transformations have involved more and more professionals from different disciplines (medicine and nutrition, but also environmental studies, biology, food studies, social anthropology, sociology, economy, etc.) who have contributed their diverse points of view on a subject that, initially, was created with only health in mind.

Interdisciplinarity is (should be) an unavoidable part of the multidimensional analysis of the Mediterranean diet. No analysis, however accurate it may be, can claim to be complete without attending to other perspectives, to other necessary categories, to other complementary questions, and to other methodologies. Different authors, such as Mackenbach [5,6], point out that beyond health sciences, humanities and social sciences are necessary to get behind why the Mediterranean diet works, and not only how it works in the body. This statement is not new and corresponds, for example, to an old claim on the part of food anthropologists [7,8,9], who argued that preventive medicine would benefit from a more holistic approach to nutritional factors and conditions. There is a very important contribution to health sciences in the holistic and anthropological approach to food and nutrition, where social and cultural perspectives are included with medicine and nutritional biology in the study of epidemiological diversity and medical needs [10,11]. On the other hand, Agaronov [12] suggests that keeping research on the Mediterranean diet relevant means partnering epidemiology with new disciplines, while parting with others, making culture “the third leg” of the epidemiological stool [13].

## 2. Scope of this Special Issue

The perspectives on the above issues are put into practice (or directly discussed) in several of the contributions to this project collection. The points of view and the contributions of the different articles that make up this Special Issue are very diverse, although they share an interest in the Mediterranean diet as the main axis. In them, the points of view from different disciplines, ranging from nutrition to medicine, biology, sociology or social anthropology among others, offer perspectives as different as they are complementary on this broad and multidimensional subject.

As in any food system, the journey regarding the Mediterranean diet begins in relation to its productive landscapes [14]. As Tomé [15] pointed out when talking specifically about Spain, the patrimonialization, popularization, and globalization of a certain conception of the Mediterranean diet have turned it into a de-territorialized global phenomenon. As a consequence of this process, it has been necessary to notably increase the production of its ingredients to satisfy its growing demand in some Mediterranean environments. This author shows in his text how the continuity of certain cultural landscapes linked to local knowledge and particular lifestyles has been sometimes broken, replacing them with agro-industrial landscapes at the service of production, causing also social and environmental inequalities.

An important part of these *Mediterranean foods* is produced for export [16]. From the perspective of health, the Mediterranean diet can also be seen as an artificially exportable item that can increase the health of different societies around the world, even saying that “the modernized Mediterranean diet concept opens the way to a scientifically-founded protective dietary pattern which could be independent from the Mediterranean geography, climate and cultures” [17].

However, the so-called Mediterranean diet is not simply a collection of foodstuffs or a set of ingredients, but a cultural expression, and collective (or individual) food habits are always constructed in a sociocultural manner. In this regard, as González Turmo also indicates, food choices, although depending on the person who buys and cooks in each household, are rarely free, even when they can appear to be one of the human activities where one’s own opinions and tastes have the greatest independence. Attitudes and behaviour are, in fact, conditioned by factors of cultural experience, which are outside the voluntary will of each of us [18].

In this regard, Scannell et al. [19] analyze, in their article in this Special Issue, the eventual transferability of the Mediterranean diet to non-Mediterranean populations such as Australia, exposing the difficulties in transplanting a foreign model to a society with different dietary patterns. They state that the perceived health benefits and improved diet quality are identified as major advantages to this eventual adoption. In contrast, dietary adherence is perceived as an important disadvantage, and self-perceived barriers are considered very relevant.

However, despite the evident and unavoidable link between food and culture, not really much has been studied about how diet contributes to the well-being of the population. In their article, Cabiedes et al. [20] analyze, from a sociological perspective, the association between subjective well-being and the eating habits of the Spanish population, in order to gain a better understanding of the subjective well-being that food culture produces. Their findings show also how perceived health and income play a key role in evaluating subjective well-being. In turn, several variables related to lifestyle habits, such as social interaction around meals, exercising, eating some specific tasty products or a low body mass index, were also associated with subjective well-being.

As we have seen in this text, social interaction around meals is one of the aspects that influences perceived health, and also subjective well-being. Conviviality (a concept that includes social interaction around meals, and it is also related to commensality) is the main subject addressed in the article by De la Torre-Moral et al. [21], and relates to *how* we eat and to the fact of sharing meals with significant people. Given the lack of research on convivial family meals in Mediterranean countries [22,23,24], this article studies family meal representations and practices of families with adolescents to assess whether they responded to a pattern of conviviality, and to examine their association with Mediterranean diet adherence. The findings of this research showed that parents believed family meals are a space for socialization and communication, but also a strategy to promote healthy eating among adolescents, having also a protective role against obesity.

The social aspects of obesity are the focus of the text by Medina et al. [25]. Obesity is a disease that straddles medico-nutritional, psychological, and socio-cultural boundaries [26,27,28,29,30]. This article presents a view of obesity in the Mediterranean context from an open, mainly socio-cultural perspective, seeking points of convergence and elements that contribute to the understanding of and approach to this disease in the context of the Mediterranean diet [31]. As a public health and a multidimensional social problem, obesity must be dealt with in a holistic, open, and cross-disciplinary manner to ensure that it can be understood coherently. The only way to keep the usefulness of the Mediterranean diet within desirable limits will be our societies’ vitality and interest in rapidly adapting the Mediterranean diet to social change, thus providing valid answers to today’s needs.

Finally, the last of the studies published in this collection [32] is the result of a preliminary study on the impact of olive oil supplement intake after strenuous physical exercise. The results show that an extra-virgin olive oil (a very central element of the Mediterranean diet) supplement could reduce the inflammatory impact of intense aerobic effort and improve recovery at 24 h.

## 3. The Need for Cross-Disciplinary Views

Human nutrition is far from being a one-dimensional affair. As the British anthropologist Mary Douglas [33] stated more than forty years ago, no human activity more puzzlingly crosses the divide between nature and culture than the selection of food. It is part of the nurture of the body, but it is also very much a social and cultural matter. Its complexity requires different views, different perspectives and different levels of comprehension. So, as some authors (mainly from social disciplines) [7,10,11,34] suggested, a cross-disciplinary approach is required, one that enables an integrative and comprehensive perspective.

The history of the Mediterranean diet concept is relatively recent yet very intense, but we shall not go into that here. All we want to underscore is that it has led to the parallel development of the following two explanatory discourses: one that is sociocultural and another that is medical–nutritional. Each of the two discourses responds to very different interests and, until fairly recently, it was nigh on impossible for them to engage in an open, productive dialogue. UNESCO’s designation of the Mediterranean diet as Intangible Cultural Heritage of Humanity in 2010 (and the preparation of the nomination in previous years) was a unique opportunity for there to be a dialogue between the two perspectives [35,36].

It is crucial for nutrition education to include some paradigm changes and for the social, economic and cultural reality of its protagonists to be taken into account to improve adherence to the practices proposed. As I previously said, the only way to keep the usefulness of the Mediterranean diet within desirable limits will be our societies’ vitality and interest in rapidly adapting the Mediterranean diet to social change, thus providing valid answers to today’s needs. The inclusion of interdisciplinary perspectives and their interpretation from open and uninterested perspectives should enrich definitions, and allow future collaborations between professionals from different disciplines and within a highly changing (evolving) sociocultural framework.
